# Supplementary material for: Causal Relationship between Aging and Anorexia Nervosa: A White-Matter-Microstructure-Mediated Mendelian Randomization Analysis
Source: Biomedicines. 2024 Aug 16;12(8):1874. doi: 10.3390/biomedicines12081874 (PMC11351342; doi:10.3390/biomedicines12081874)
Supplement: Supplementary file 1 [file biomedicines-12-01874-s001.zip › Supplementary Figure/Figure S7/Single SNP plot of MR analysis for ubm-b-1961 to AN.pdf]

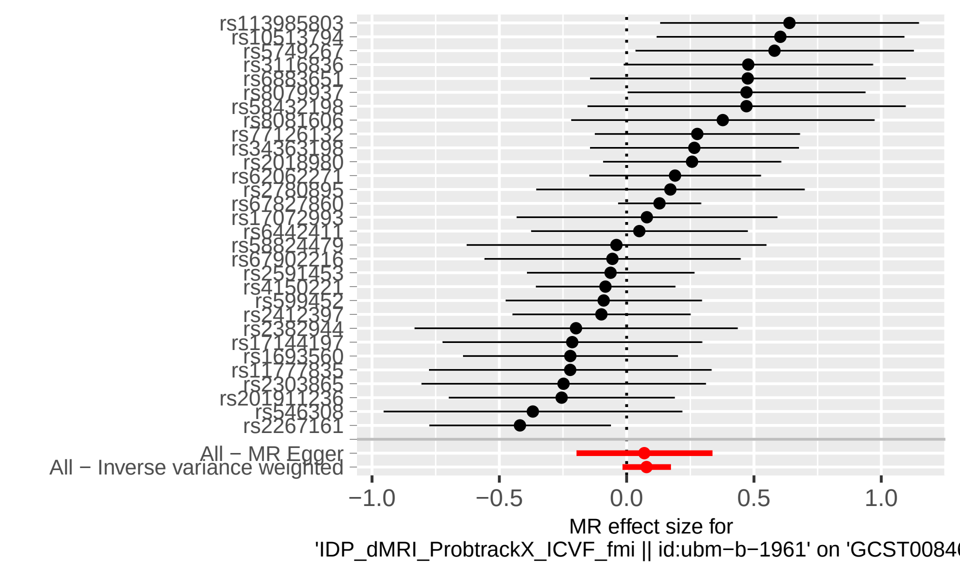

rs113985803
rs10513794
rs5749267
rs3116836
rs6883651
rs8079937
rs58432198
rs8081606
rs77126132
rs34363198
rs2018980
rs62062271
rs2780895
rs67827860
rs17072993
rs6442411
rs58824479
rs67902216
rs2591453
rs4150221
rs599452
rs2412397
rs2382944
rs17144197
rs1693560
rs11777835
rs2303865
rs201911236
rs546308
rs2267161
All − MR Egger
All − Inverse variance weighted
−1.0
−0.5
0.0
0.5
1.0
MR effect size for
'IDP_dMRI_ProbtrackX_ICVF_fmi || id:ubm−b−1961' on 'GCST0084
